# Complete genome sequence of *Saccharolobus islandicus* strain DK8, a thermoacidophilic archaeon from Lassen Volcanic National Park

**DOI:** 10.1128/mra.00739-26

**Published:** 2026-08-19

**Authors:** Jonathan Abshier, Kenneth Stedman

**Affiliations:** 1 Department of Biology and Center for Life in Extreme Environments, Portland State University 6685, Portland, Oregon, USA; University of Wisconsin-Madison, Madison, Wisconsin, USA

**Keywords:** archaea, extremophiles

## Abstract

Here, we report the genome sequence of *Saccharolobus islandicus* strain DK8, a thermoacidophilic archaeal isolate from the Devil’s Kitchen geothermal area of Lassen Volcanic National Park, California, USA. The genome is 2,783,064 bp, with a guanine-cytosine content of 35.02%. Strain DK8 is most closely related to *Sulfolobus islandicus* L.S.2.15.

## ANNOUNCEMENT

Thermoacidophilic archaea of the order Sulfolobales inhabit acidic, high-temperature geothermal environments and are important models for studies of archaeal molecular biology, genome evolution, and virus-host interactions (1–5). *Saccharolobus islandicus* has also been used as a model for studying archaeal species boundaries, gene flow, and virus-associated genome variation across geographically separate hot springs (2, 3). Genome resources from geographically related isolates are useful for examining local archaeal diversity and determinants of virus susceptibility.

*S. islandicus* strain DK8 was isolated from a hot spring in the Devil’s Kitchen geothermal area of Lassen Volcanic National Park, California, USA “Troll Spring” at 40°26.469′ N, 121°26.061′ W on 20 July 2024. The sample had an approximate pH of 1.5 to 2 and a temperature of 77°C to 78°C. This is the same location that *S. solfataricus* strain S441 was isolated from (6). Strain DK8’s genome was sequenced to provide a comparative genome resource.

The environmental sample was processed using the same isolation and growth procedures previously used for Sulfolobales isolates (6). Briefly, the turbid water and sediment sample was adjusted to approximately pH 5 with solid calcium carbonate on site and transported anaerobically at ambient temperature to Portland, Oregon. The sample was enriched aerobically in Sulfolobus medium (7) at 80°C for 6–7 days. Strain DK8 was isolated as a single colony by plating on 1% Gelrite in the same media. For DNA preparation, DK8 was grown from frozen stocks in Sulfolobus medium at pH 3.5 and 80°C. Chromosomal DNA was extracted using TEN buffer, Triton X-100, phenol-chloroform-isoamyl alcohol extraction, and ethanol precipitation (6–8).

DNA was sequenced using Oxford Nanopore Technologies (ONT). Library preparation used the ONT Rapid Barcoding Kit V14, and sequencing used an R10.4.1 flowcell on a GridION instrument running MinKNOW. Base calling used Dorado v0.7.4. No size selection was performed prior to sequencing. Post-sequencing read filtering used SeqKit to remove reads with quality scores below Q7 (9). ONT sequencing generated 117,022 reads totaling 798,062,401 bases, with a mean read length of 6,819.76 bp, and read N50 of 13,079 bp. The genome was assembled *de novo* using Flye v2.9.6 with an estimated size of 2.7 Mb (10). The final assembly consisted of one contig totaling 2,730,524 bp, a guanine-cytosine (GC) content of 35.02%, no ambiguous bases, and an estimated mean coverage depth of 289×. Bandage v0.9.0 was used to visualize the Flye assembly graph (11). The genome was not manually rotated. The assembled genome was annotated with the National Center for Biotechnology Information (NCBI Prokaryotic Genome Annotation Pipeline (PGAP v6.11) (12). For details see Table 1.

**TABLE 1:** Genome assembly and annotation summary for *Saccharolobus islandicus* strain DK8

Feature	DK8
Genome length (bp)	2,783,064
Number of contigs	1
Assembly N50 (bp)	2,783,064
Mean genome coverage	289×
GC content (%)	35.02
Total genes	3,512
Total CDSs	3,469
rRNAs	3
rRNA types	5S, 16S, 23S
tRNAs	38
ncRNAs	2
CRISPR arrays	7

Pairwise average nucleotide identity (ANI) analysis was conducted using FastANI (13) to compare strain DK8 to *Saccharolobus solfataricus* strain S441, CP160827.2 (87.49%), isolated from the same location, *Sulfolobus islandicus* strain REY15A CP002425 (96.79%), and *Sulfolobus islandicus* strain L.S.2.15 CP001399 (98.06%).

The DK8 genome expands the available sequence resources for Sulfolobales isolates from Lassen Volcanic National Park. Together with the genome of *S. solfataricus* strain S441, DK8 provides a local comparator for studies of archaeal genome evolution, CRISPR spacer content, integrated mobile elements, and virus-host interactions in acidic geothermal environments.

## Data Availability

The genome sequence of *Sulfolobus islandicus* strain DK8 has been deposited in GenBank under accession number JBZAYF000000000. The associated BioProject, BioSample, and SRA raw sequencing reads are available under accession numbers PRJNA1476781, SAMN60731919, and SRP708253, respectively. Strain DK8 is available from the authors on request.
